# A Case Report of Cold Agglutinin Disease, Severe B12 Deficiency, and Pernicious Anemia: A Deadly Coincidence

**DOI:** 10.7759/cureus.38208

**Published:** 2023-04-27

**Authors:** Nehemias A Guevara, Esmirna Perez, Jorge Sanchez, Flor Rosado, Harry G Sequeira Gross, Ilmana Fulger

**Affiliations:** 1 Internal Medicine, St. Barnabas Hospital Health System, Bronx, USA; 2 Internal Medicine, Department of Hematology-Oncology, St. Barnabas Hospital Health System, Bronx, USA

**Keywords:** critical anemia, severe anemia, pernicous anemia, vitamin b12 deficiency anemia, cold agglutinin disease

## Abstract

Anemia is the most common hematological disorder. It is commonly a manifestation of an underlying disease. Its causes are multifactorial, including but not limited to nutritional deficiencies, chronic conditions, inflammatory processes, medications, malignancy, renal dysfunction, hereditary diseases, and bone marrow disorders. We present a case of a patient exhibiting anemia related to cold agglutin disease and severe B12 deficiency secondary to pernicious anemia.

## Introduction

Anemia is a condition where the red cell number is reduced. Hemolytic anemia is present when the reduction in the quantity of RBC is caused by decreased cell lifespan secondary to cell destruction. The causes of hemolytic anemia include immune hemolytic disorders, hemoglobin disorders, RBC enzyme disorders, and RBC membrane disorders [1,2]. Clinical presentation is determined by decreased RBC number and rapid cell turnover metabolic consequences. Common presenting signs and symptoms include pallor, episodes of jaundice, splenomegaly, and gallstones. Patients may or may not have a history of blood transfusions and a family history of anemia [3].

Hemolytic anemias can be part of presenting specific syndromes. One spectrum of hemolytic anemia is thrombotic microangiopathy syndrome (TMA), which involves microangiopathic hemolytic anemia, thrombocytopenia, and organ damage. Causes of TMA are diverse, all leading to endovascular damage triggering hematologic manifestations. One of the described causes of TMA or pseudo-TMA is vitamin B12 deficiency [3,4]. Uncommon but under the spectrum of hemolytic anemia is cold agglutinin disease (CAD), which is a rare presentation in which cold agglutinins (IgM autoantibodies against RBC antigens with an optimum temperature of 3 to 4°C) cause agglutination in cooler parts of the body [5,6].

 Vitamin B12 is a water-soluble vitamin essential for DNA synthesis, formation, and maturation of hematopoietic cells and for preserving normal neurological function. Hemolytic presentation of vitamin B12 deficiency has been related to hyperhomocysteinemia through reactive oxygen species generation leading to membrane damage, endothelial cell compromise, and platelet aggregation with consequent thrombotic microangiopathy. Recognizing this entity in severe vitamin B12 deficiency is crucial, as TMA could be life-treating if not promptly managed [3,4]. We report a case of a patient who presented with severe vitamin B12 deficiency secondary to malnutrition and pernicious anemia but was also found to have CAD. We review the approach to diagnosis and management.

## Case presentation

A 44-year-old male with a past medical history of alcohol use disorder was brought into the hospital by emergency medical services with complaints of fatigue and yellow skin discoloration. As per the patient, he started having intermittent abdominal pain with anorexia and diarrhea about five months before presentation with a reported 20 lbs weight loss. Symptoms worsened with fatigue, nausea, vomiting, dark urine, and jaundice one week before the presentation. The patient states that his diet was primarily liquid, predominantly sodas, during this time. The patient denied any other accompanying symptoms, drug use, recent infections, travels, or risk of sexual activity.

On physical examination, profound pallor and mild jaundice of skin and mucosae were noticed, with no peripheral lymphadenopathy. The abdomen has mild right upper quadrant tenderness at palpation without hepatosplenomegaly or abdominal masses. Laboratory workup was remarkable for pancytopenia with severe anemia, high reticulocyte count, mild transaminitis, elevated lactate dehydrogenase (LDH), and hyperbilirubinemia with indirect bilirubin predominance (Table 1).

**Table 1:** Initial workup MVC: mean corpuscular volume, ALT: alanine transaminase, AST: aspartate aminotransferase, INR: international normalized ratio, LDH: lactate dehydrogenase

Variable	On admission	Reference range
WBC count	2.0	4.2-9.1 10*3/uL
Hemoglobin	2.5	13.7-17.5 gm/dL
Hematocrit	6.6	40.1-51.0%
MCV	117.9	79.0-92.2 fL
Platelet count	29	150-450 10*3/uL
Reticulocyte	1.92	0.51-1.81 %
Immature reticulocyte	20.0	2.3-13.4%
Reticulocyte absolute	44.9	24.1-35.8 pg
ALT	127	4-36 IU/L
AST	105	8-33 IU/L
Direct bilirubin	0.7	0.0-0.3 mg/dL
Bilirubin total	4.0	0.1-1.2 mg/dL
INR	1.3	0.9-1.1
Calcium	7.9	9.2-11.0 mg/dL
Albumin	3.1	3.8-5.0 gm/dL
LDH	1297	100-190 IU/L
Vitamin B12	6	160=950 pg/mL
Folic acid	19.7	3.0-999.0 ng/mL

On peripheral blood smear (PBS), there was decreased number of RBC with RBC agglutinates, anisopoikilocytosis with dacrocytes, rare schistocytes, without polychromasia, nucleated RBC, associated with decreased number of WBC with some hyper segmented neutrophils. There were no toxic granulations or clumps but decreased platelets with occasional large and no platelet clumps. Blasts were not observed.

Direct Coombs test was positive with elevated cold agglutinin titers. Furthermore, there were positive parvovirus IgG and IgM, low complement levels (C3 and C4), and direct Coombs. However, the immune workup was negative. The hematology service evaluated the patient. Profound pancytopenia was considered multifactorial, with nutritional etiology very likely in the setting of alcohol abuse and poor nutrition. Therefore, the patient was managed with warm blood transfusions, a total of three units of packed RBC, and high doses of steroids, a suspicion of CAD.

**Figure 1:**
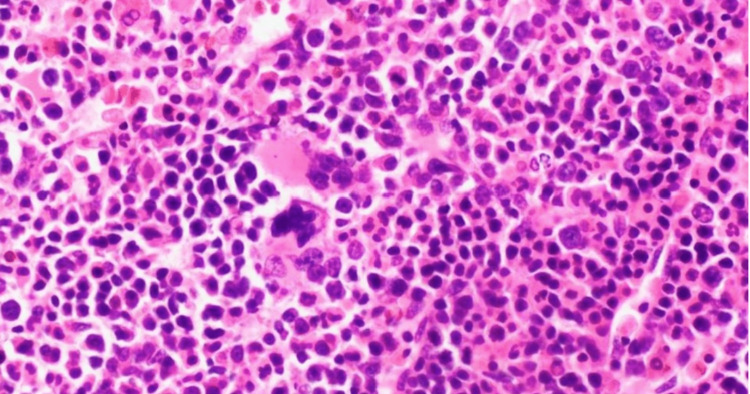
Bone marrow aspirate and core biopsy with findings of hypercellular marrow (90%) showing erythroid hyperplasia, erythroid, and megakaryocyte atypia. Flow cytometry showed no immunophenotypic evidence of a lymphoproliferative disorder or a plasma cell neoplasm. Findings are consistent with the patient's history and vitamin B12 deficiency

Further workup was done. The patient was found to have severe B12 deficiency with a serum vitamin level of 8 pg/mL. Therefore, an intensive supplementation was initiated with intramuscular cyanocobalamin 1000 mcg injections daily for a total of seven days. Free light chains were elevated in the serum and urine. No M spike was observed in the serum; however, an M spike was present in the urine. Anti-parietal and anti-intrinsic factor antibodies were positive. In light of the findings, a bone marrow biopsy was performed (Figure 1).

The patient showed progressive improvement and appropriate response to transfusions. The patient was discharged on oral steroids with almost complete resolution of symptoms. On subsequent evaluation after 12 weeks, the patient was noticed to have resolved pancytopenia and complete resolution of symptoms. The patient was discharged on oral steroids with almost complete resolution of symptoms. On subsequent evaluation after 12 weeks, the patient was noticed to have resolved pancytopenia and complete resolution of symptoms.

## Discussion

Anemia is the most common hematological disorder. Its causes are multifactorial, including but not limited to nutritional deficiencies, chronic conditions, inflammatory processes, medications, malignancy, renal dysfunction, hereditary diseases, and bone marrow disorders [7,8].

It can be categorized based on the sizes of the erythrocytes, better known as mean corpuscular volume (MCV) in microcytic, normocytic, or macrocytic anemia. If MCV is < 80 femtoliters (fL), 80 - 100 fL, or > 100 fL, respectively [9]. Hemolysis, the destruction of RBC [6], is one of the multiple causes of anemia. It can be further classified as intrinsic to the erythrocytes (membrane, enzyme, or hemoglobin defects) or extrinsic to the erythrocytes (immune-mediated, infectious, or due to thrombotic microangiopathy) [7,10].

Hemolytic anemias should be in the differential diagnosis for any normocytic or macrocytic anemia [10] and can present clinically as a patient with fatigue, weakness, jaundice, and hematuria (dark urine). However, laboratory evidence is essential for the definitive diagnosis, including but not limited to an increase in reticulocyte count (representing bone marrow regenerative effort), an elevated LDH (translated as increased cell destruction), and an elevation in unconjugated bilirubin (increased hemoglobin catabolism) [7,10], all of which were present in our case. It is important to note that it is beneficial to distinguish if the hemolysis is happening within the blood vessels (intravascular) or out of the blood vessels (extravascular, in organs such as the spleen and liver) [7,11] and how essential the PBS is to aid in separating the two [12]. When suspecting intravascular hemolysis, schistocytes are expected in the PBS [13], just like in this case.

Microangiopathic hemolytic anemia, also known as thrombotic microangiopathy, is one of the causes of extravascular hemolysis. It has several etiologies; however, vitamin B12 deficiency as the cause is infrequent. It can be mistakenly diagnosed as another cause of thrombotic disease such as thrombocytopenic purpura (TTP), as it did in the case of Osman et al. [3], hence the importance of analyzing vitamin B12 levels in this type of case. Vitamin B12 deficiency has three main etiologies: autoimmune, malabsorption, and dietary insufficiency [14]. Our case presented had a combination of two: autoimmune secondary to pernicious anemia, which was found during the admission, and dietary insufficiency due to alcoholism. Cases of severe vitamin B12 deficiency mimicking TMA have been reported in the past [15-17], but none as severe as the case presented today, with a vitamin B12 level of 6 and a positive direct Coombs test with RBC agglutination.

The direct Coombs test tends to be negative; however, in our case, the direct Combs test was positive, making us believe autoimmune anemia might be involved. Nonetheless, cases of transient direct Coombs positive test on TMA have been reported, as was the case of Dan et al. [18]; a transient positivity can be observed in up to 90% of patients with TMA secondary to neuraminidase-producing Streptococcus pneumonia [18], as well as the case of Zenno and Richardson [19], where a patient with TTP was found to have a positive Coombs test, which is a rare phenomenon [19].

The mechanism of how hemolysis develops is not completely understood; it is believed to be due to the increase in metabolites depending on vitamin B12 as a cofactor for their reaction, such as methylmalonic acid and homocysteine. The increased levels of homocysteine and subsequent accumulation produce hemolysis by oxidative damage. However, this is extremely rare in only 1.5% of severe deficiencies [20-22].

The RBC agglutination observed in this case broadens and strengthens the possibility of the hemolysis anemia not only being related to vitamin B12 deficiency but having an autoimmune etiology as well, specifically CAD, making it a fatal combination, worsening the presentation and the degree of hemolysis. Hemolysis in CAD occurs secondary to the binding of cold agglutinin IgM to the RBC surface, activating the complement cascade, forming a unit that is later destroyed by macrophages present predominantly in the liver and spleen [23, 24]. CAD and vitamin B 12 deficiency have been linked in the past, as were the cases presented by Imashuku et al. [25], where the emphasis was made on the association between autoimmune hemolytic anemia and megaloblastic anemia and the importance of concomitant testing.

There also have been cases in which severe vitamin B 12 deficiency presents as CAD until further testing is performed, such as the case of De La Puerta et al. [26], where the deficiency was mistaken as CAD given the presentation of hemolytic anemia with supportive laboratories markers, as well as with the case presented by Cikrikcioglu et al. [27], where the vitamin B12 deficiency first presented as hemolysis anemia. However, their patient had thalassemia minor. Subsequently, macrocytosis was absent in PBS, making the diagnosis more convoluted.

In our case, the patient had a negative immune workup, low complement levels, and direct Coombs positive with IgM/IgG parvovirus, which is one of the most common triggers of CAD [24]. CAD is a diagnosis of exclusion, of rare presentation, but even rarer is having three ongoing medical conditions simultaneously.

## Conclusions

It is of utmost importance to associate hemolytic anemia with vitamin B12 deficiency and to be aware that the most severe presentations of the deficiency can mimic several blood disorders. When keeping this in mind, the clinician can ensure early diagnosis, which will prompt early diagnosis, appropriate treatment, faster recovery, and avoidance of unnecessary and sometimes hurtful tests.
